# MST1/2 inhibitor XMU‐MP‐1 alleviates the injury induced by ionizing radiation in haematopoietic and intestinal system

**DOI:** 10.1111/jcmm.17203

**Published:** 2022-01-27

**Authors:** Xiaoliang Zhou, Hao Wang, Deguan Li, Naling Song, Fujun Yang, Wenqing Xu

**Affiliations:** ^1^ Tianjin Key Laboratory of Radiation Medicine and Molecular Nuclear Medicine Institute of Radiation Medicine Chinese Academy of Medical Science & Peking Union Medical College Tianjin China; ^2^ Academy of Medical Engineering and Translational Medicine Tianjin University Tianjin China

**Keywords:** haematopoietic injury, hippo pathway, ionizing radiation, radiation enteritis, XMU‐MP‐1

## Abstract

The Hippo signalling pathway has been considered as potential therapeutic target in self‐renewal and differentiation of stem and progenitor cells. Thus, mammalian Ste20‐like kinase 1/2 (MST1/2) as the core serine‐threonine kinases in the Hippo signalling pathway has been investigated for its role in immunological disease. However, little information of MST1/2 function in bone marrow suppression induced by ionizing radiation was fully investigated. Here, we reported that MST1/2 inhibitor XMU‐MP‐1 could rescue the impaired haematopoietic stem cells (HSCs) and progenitor cells (HPCs) function under oxidative stress condition. Also, XMU‐MP‐1 pretreatment markedly alleviated the small intestinal system injury caused by the total body irradiation 9 Gy and extended the average survival days of the mice exposed to the lethal dose radiation. Therefore, irradiation exposure causes the serious pathological changes of haematopoietic and intestinal system, and XMU‐MP‐1 could prevent the ROS production, the haematopoietic cells impairment and the intestinal injury. These detrimental effects may be associated with regulating NOX/ROS/P38MARK pathway by MST1/2.

## 1 INTRODUCTION

There are constant research, ongoing innovation and technical and scientific advancements in order to tackle different forms of cancer. Various molecules have been considered as the therapeutic targets for cancer prevention.^1^, ^2^, ^3^, ^4^, ^5^, ^6^, ^7^ Excluding chemotherapy, radiation therapy is another cancer treatment that uses high doses of radiation to kill cancer cells and shrink tumours. However, ionizing radiation can induce irreversible damage in all radiosensitive organs. The level of damage depends on the radiation dose absorbed, radiation type and organ sensitivity. The haematopoietic system is most sensitive to radiation, especially the HSCs undergo senescence *in vitro* and *in vivo* following irradiation exposure.^8^, ^9^ Therefore, the bone marrow suppression would be observed by encompassing all cell lines causing anaemia, leucopenia, thrombocytopenia and neutropenia.^10^ The ROS production is one crucial reason for radiation damage; furthermore, the persistent oxidative stress is observed in haematopoietic stem/progenitor cells (HSPCs). Previous researches revealed the persistent ROS elevation induced oxidative stress associated with the activation of NOX4 signalling.^11^, ^12^ NOX4 mediates ROS production in radiation‐induced senescent cells and contributes to normal tissue damage after IR via the recruitment of inflammatory cells and the exacerbation of tissue inflammation. In addition, radiation‐induced ROS production was diminished by genetic or pharmacological inhibition of NOX4.^13^

High‐dose irradiation (>10 Gy) can also cause severe intestinal toxicity, known as gastrointestinal syndrome. Under homeostatic conditions, rapid turnover of enterocytes is driven by ISCs located at the base of the crypts. XMU‐MP‐1 as an inhibitor of mammalian Ste20‐like kinase 1/2 (MST1/2), displayed excellent *in vivo* pharmacokinetics and was able to promote mouse intestinal repair. The Hippo pathway regulates the self‐renewal and differentiation of stem and progenitor cells and plays key roles in controlling organ size and regeneration.^14^, ^15^ MST1/2, mammalian homologs of Hippo, are a core pair of serine‐threonine kinase in the Hippo signalling pathway that regulates the cell cycle and apoptosis.^16^ A recent study showed that MST1/2 are also crucial for CD8α+ dendritic cell‐mediated antigen presentation to CD8+ T cells, which requires a balance of metabolic activity and cytokine signalling depending on MST1/2 activity.^17^ And it is reported that MST1/2 functioned to control ROS production by regulating mitochondrial trafficking and mitochondrion‐phagosome juxtaposition.^18^ Studies of MST1‐deficient mice have demonstrated that MST1 regulates the production of reactive oxygen species (ROS) through FOXO3A (forkhead box O3A), thereby preventing apoptosis of peripheral T cells.^19^ Meanwhile, the roles of MST1/2 among HSPCs *in vivo* did not reach an agreement. We hypothesized that MST1/2 could associate with the ROS production in the mitochondria of HSPCs, and XMU‐MP‐1 as a putative MST1/2 inhibitor was utilized as a novel radioprotectant to ameliorate TBI‐induced residual BM injury. The effect of XMU‐MP‐1 on the intestinal system injury induced by radiation was assessed as well.

## 2 MATERIALS AND METHODS

### 2.1 Reagents

XMU‐MP‐1 was provided by Selleck chemicals; 2′, 7′‐Di‐chlorodi‐hydrofluorescein diacetate (DCFDA) and dichloro‐fluorescein (DCF) were obtained from Sigma. Anti‐mouse Ly‐6G/Ly‐6C (Gr‐1) was abtained from BD bioscience. Anti‐mouse CD117(c‐kit)‐APC, anti‐mouse Ly‐6A/EA(Sca‐1)‐PE/Cy7, biotin‐conjugated anti‐mouse CD45R/B220, anti‐mouse CD11b, anti‐mouse Ter‐119 and APC‐Cy7‐conjugated streptavidin were obtained from eBioscience. Rabbit anti‐γ‐H2AX was obtained from Cell Signaling Technology. Rabbit anti‐NOX4 was obtained from Proteintech. FITC‐conjugated goat anti‐rabbit antibodies were obtained from Abcam Biotechnology. Cytofix/Cytoperm buffer, Perm/Wash buffer and Cytoperm Permeabilization Buffer Plus were obtained from BD Pharmingen.

### 2.2 Animals and ionizing radiation

Male C57BL/6 mice (8–10 weeks) were provided by Beijing HFK Bioscience Co. Ltd. Animals were housed in the certified animal facility (Specific Pathogen Free level) at Institute of Radiation Medicine (IRM), Chinese Academy of Medical Sciences (CAMS). All procedures involving animals were reviewed and approved by the Animal Care and Use Committee of IRM (Permit Number 1638).

Mice were randomly divided to 3 groups: (a) a control group (Ctrl); (b) irradiated group (IR); (c) radiation +XMU‐MP‐1 group (IR+XMU). The control group was administrated with the vehicle solution (normal saline including 10% DMSO), the irradiated group was applied with different dose radiation, and XMU‐MP‐1 (10 mg/kg) dissolved in vehicle solution was injected intraperitoneally to mice in the IR+XMU group for 7 days prior to radiation.

For assessing the radiation injury in the haematopoietic system, animals (*n* = 5 per group) were exposed to total body irradiation (TBI) at a single sublethal dose of 4.0 Gy and then monitored for 9 days. Regarding the radiation challenge to the small intestine, mice (*n* = 5 per group) were exposed to 9 Gy TBI, and small intestine was harvested for the histology parameter evaluation after 3.5 days. The 30 days survival assay was performed by exposing the mice (*n* = 10 per group) to the lethal dose 7.5 Gy, and then, the irradiated mice were monitored for survival through 30 days. The average survival time was calculated as follows^20^: The average survival time = SUM (survival days of mice)/mice number.

All the ionizing radiation exposure was performed at the Institute of Radiation Medicine, Chinese Academy of Medical Sciences, with a Cs^137^ γ‐radiation source (Atomic Energy of Canada Ltd) at a dose rate of 1.02 Gy/min.

### 2.3 Peripheral blood cells and bone marrow nucleated cells (BMNCs) counts

Blood samples isolated by orbital sinus were kept in the tube with ethylenediaminetetraacetic acid (EDTA) and tested by Celltac E hemocytometer (Nihon Kohden). The BMNCs were collected by flushing the femur with cold PBS and analysed with the hemocytometer.

### 2.4 Isolation of HPCs and HSCs

Haematopoietic progenitor cell and HSCs in bone marrow were sorted by the method previously reported.^21^ Bone marrow cells were suspended in phosphate‐buffered saline (PBS), and 5 × 10^6^ bone marrow cells were filtered and counted prior to antibody staining. The bone marrow cells were first stained with biotin‐labelled antibodies specific for murine CD4, CD8, CD11b, CD45R/B220, Ter‐119 and Gr‐1, and then stained with streptavidin‐APC‐Cy7, anti‐Sca‐1‐PE and anti‐c‐kit‐Alexa Fluor 700. HPCs (Lin^−^c‐kit^+^Sca‐1^−^) and HSCs (Lin^−^c‐kit^+^Sca‐1^+^) were analysed and sorted using a flow cytometer (BD Accuri™ C6 Plus).

### 2.5 ROS level analysis on HSCs, HPCs and BMNCs with irradiation exposure.

Reactive oxygen species level of HSCs, HPCs and BMNCs was tested by the flow cytometry with 2,7‐dichlordihydrofluorescein diacetate (H2DCFDA, Invitrogen), as the ROS probe after IR exposure. BM lineage negative haematopoietic cells (Lin–cells) were stained with anti‐Sca‐1‐PE and anti‐c‐kit–Alexa Fluor 700 antibodies and then added with 5 μM H2DCFDA and incubated for 30 min at 4°C. Cells were washed twice with PBS and resuspended in FACS buffer (PBS +0.1% FBS). Mean fluorescence intensity (MFI) was measured on a flow cytometer (BD Accuri™ C6 Plus).

The HSCs protein expression level of NOX4, γ‐H2AX, p‐P38 and Nrf2 was evaluated by the flow cytometry. Bone marrow cells were first stained with a c‐kit antibody and fixed and permeabilized with Cytofix/Cytoperm buffer (BD Biosciences, USA) before being stained with the primary antibody including anti‐NOX4^22^ (14347‐1‐AP, Proteintech), γ‐H2AX^23^ (Cell Signaling Technology), anti‐p‐P38 antibody^24^ (BD Pharmingen) (1:50), anti‐Nrf2^23^ (ab62352, Abcam) at room temperature. The diluted secondary antibodies were added and incubated at room temperature. Then, the cells were resuspended in FACS buffer and measured on a flow cytometer (BD Accuri™ C6 Plus).

### 2.6 The immune cells proportion analysis of the peripheral blood

For analysis of multilineage blood cells, FITC‐Anti‐GR‐1 (BD), FITC‐Anti‐CD45R/B220 and FITC‐Anti‐CD11b antibodies were used for the test of the peripheral blood. And the proportion of positive marked cells was measured on a flow cytometer (BD Accuri™ C6 Plus).

### 2.7 Histological analysis

Mice were sacrificed, and jejunum was isolated after TBI 3.5 days, fixed overnight in 10% neutral formalin and embedded in paraffin. 5‐μm sections were prepared and stained with haematoxylin and eosin (H&E) according to standard protocols and observed under a light microscope (Olympus Corp. Japan).

### 2.8 Organ weight index detection

Animals were sacrificed organs including the thymus, spleen, heart, kidney, lung and liver were isolated. Organ index was calculated using the following equation: Organ index = organ weight/body weight.

### 2.9 Statistical analysis

The results were expressed as the mean ± SD. An unpaired *t*‐test (two‐tails) was used for the majority of comparisons. Significant differences between experimental groups were evaluated using one and two‐way analysis of variance (ANOVA) with repeated measures followed by post hoc comparisons with Tukey's multiple paired comparison test. Significance thresholds of *p < 0.05, **p < 0.01 and ***p < 0.001 were applied. All analyses were performed using GraphPad Prism software.

## 3 RESULTS

### 3.1 XMU‐MP‐1 rescues the damage of the haematopoietic system

**FIGURE 1:**
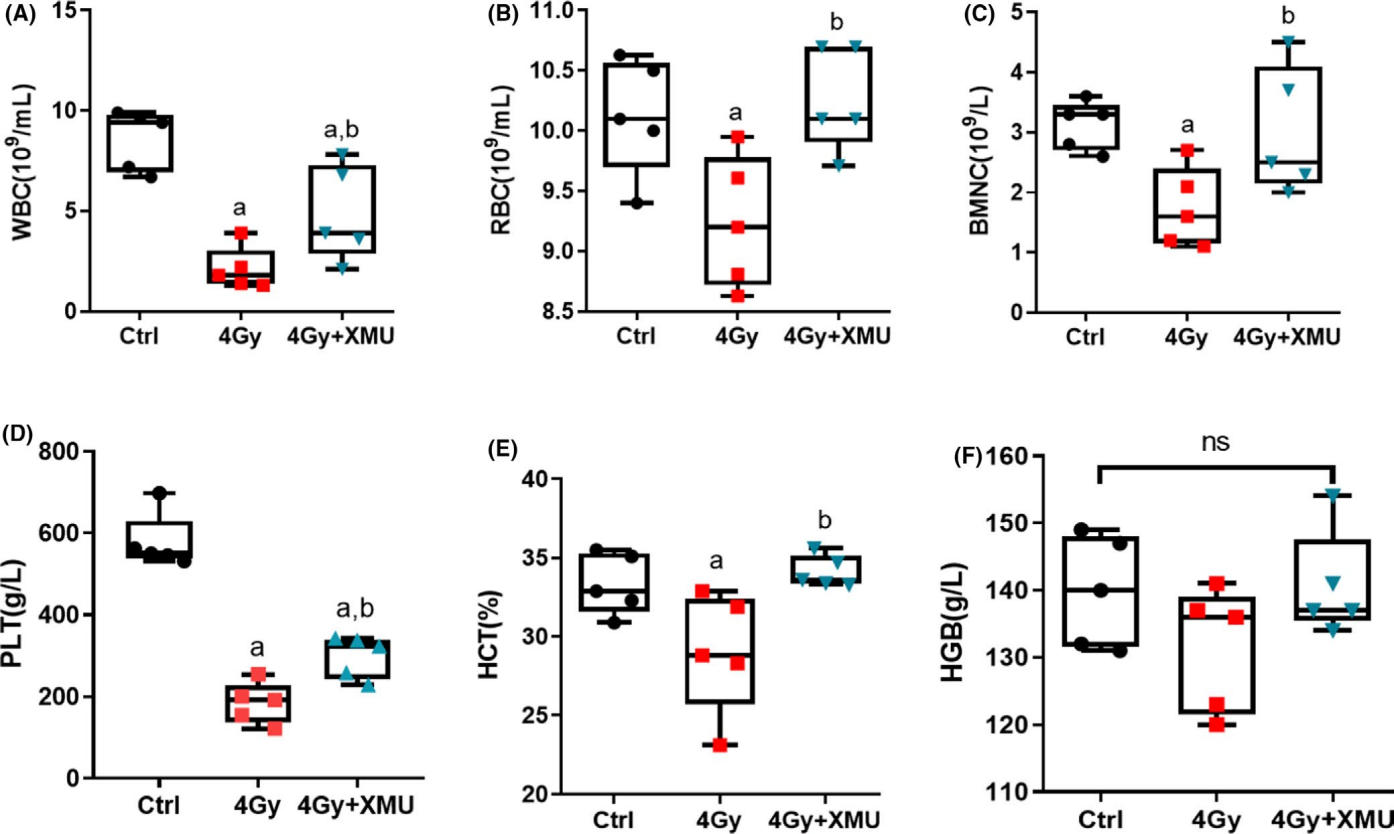
XMU‐MP‐1 rescues the damage induced by IR on the haematopoietic system. Mice were administrated with XMU‐MP‐1 (10 mg/kg) intraperitoneally for 7 days before 4 Gy IR and blood samples were collected 9 days after IR. The cells counting of WBC(A), RBC(B), BMNCs and the level of HCT, PLT, HGB were tested. Results are expressed as Mean ± SD, *n* = 5 per group. (Significance: ^a^ *p* < 0.05 vs Ctrl; ^b^ *p* < 0.05 vs 4 Gy). WBC (White blood cell); RBC (Red blood cell); HCT (haematocrit); PLT (platelet); HGB (haemoglobin); BMNC (bone marrow nucleated cell)

Haematopoietic system is vulnerable to ionizing radiation, especially, WBC as a sensitive marker of irradiation effect is associated with the irradiation dose and time. The results (Figure 1) show that the WBC of the mice exposed to 4 Gy TBI decreased to 24.7% of that in the control group (*p* < 0.001). XMU‐MP‐1 could significantly rescue the WBC impairment after IR by increasing the WBC to 56.4% of pre‐irradiation (*p* < 0.05). The effect of radiation exposure on RBCs, HCT, PLTs and BMNCs showed the same pattern with the WBC, while the HGB level was not obviously influenced at 9 days after IR.

### 3.2 XMU‐MP‐1 reduces the ROS level of haematopoietic system

**FIGURE 2:**
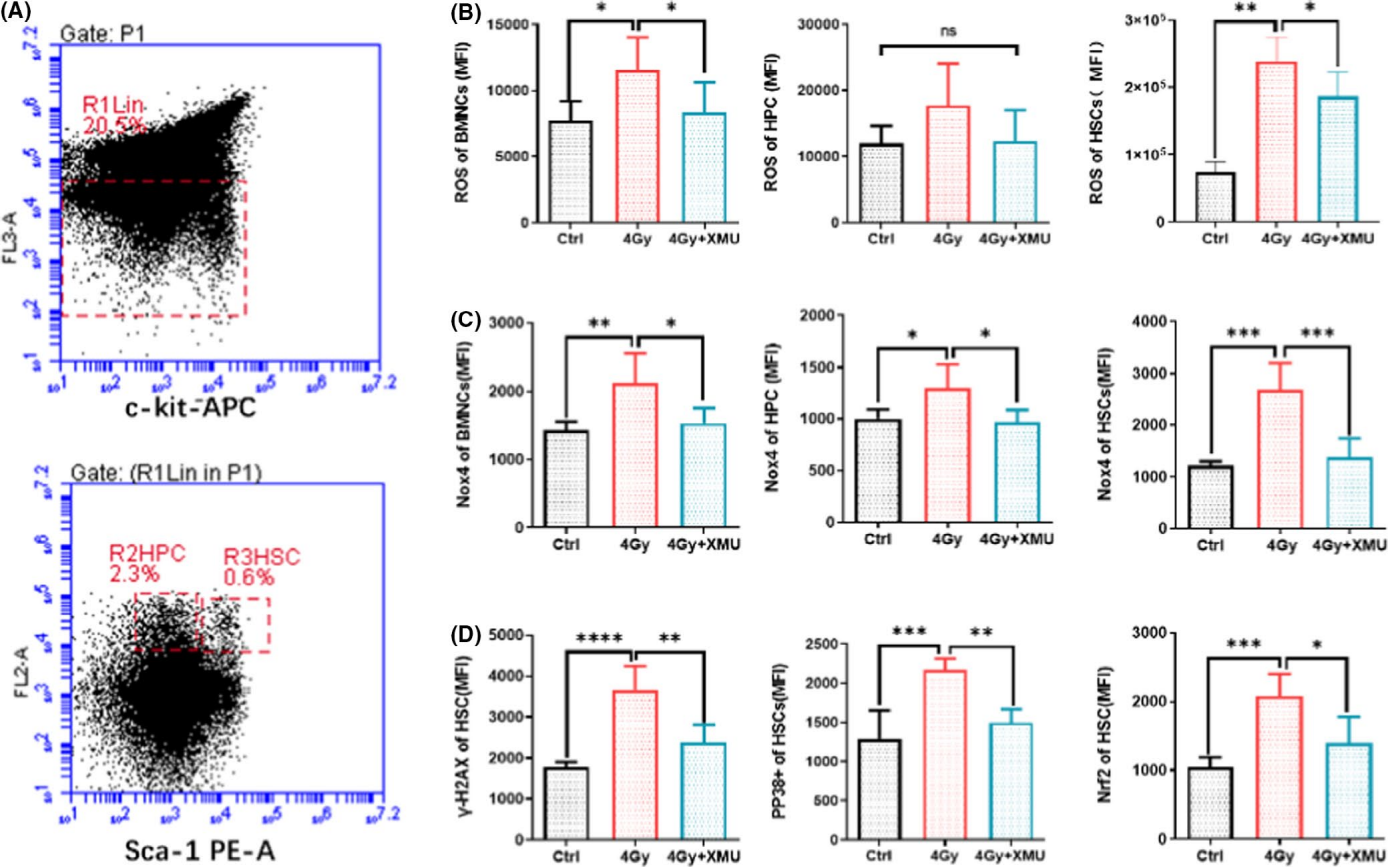
XMU‐MP‐1 ameliorates ROS induced by IR on the haematopoietic system. Mice were administrated with XMU‐MP‐1 for 7 days before being exposed to TBI 4 Gy, and samples were harvested 9 days after IR. (A) A representative gating strategy of HSC and HPC analysis by flow cytometry; (B) ROS level of BMNCs, HPCs and HSCs; (C) NOX4 expression level of BMNCs, HPCs and HSCs; (D) The γ‐H2AX, PP38+ and Nrf2 level of HSCs. Results are expressed as Mean ± SD, *n* = 5 per group. (Significance: *p < 0.05, **p < 0.01 and ***p < 0.001)

Radiation generates ROS that can interact with a various cellular molecular including DNA, lipids and proteins. To determine if the effect of XMU‐MP‐1 on haematopoietic system, we assessed the ROS level of BMNCs, HPCs and HSCs. The intracellular ROS level was measured by H2DCFDA in irradiated mice upon XMU‐MP‐1 treatment. As Figure 2A shown, the R1 gate was to measure the lineage negative cells, while the HPCs and HSCs were gated by R2 (lineage‐Sca‐1‐c‐Kit+) and R3 (lineage‐Sca‐1+c‐Kit+), respectively. The result (Figure 2B) shows that ROS increased significantly (BMNCs 1.5‐fold, *p* < 0.05; HSCs 3.2‐fold, *p* < 0.01) after radiation, while XMU‐MP‐1 pretreated for 7 days before irradiation could obviously relieve ROS accumulation to 72% (BMNCs, *p* < 0.05) and 78% (HSCs, *p* < 0.05) separately compared with irradiation alone. While ROS level in HPCs of the irradiated mice shows the similar pattern, but the difference is not significant. NOX4, as the phagocyte‐type oxidase, is responsible for the production of ROS. As Figure 2C shown that the NOX4 expression of the mice exposed to 4 Gy TBI was induced obviously to 1.5‐fold (BMNCs, *p* < 0.05), 1.3‐fold (HPCs, *p* < 0.05) and 2.2‐fold (HSCs, *p* < 0.001) compared with the control groups. And NOX4 expression associated with ROS induction could be significantly decreased by XUM‐MP‐1 pretreatment, which recovered to 72% (BMNCs, *p* < 0.05), 74% (HPCs, *p* < 0.05) and 52% (HSCs, *p* < 0.001) compared with 4.0 Gy TBI. Additionally, the HSCs protein expression of γ‐H2AX, Nrf2 and PP38 was measured in the mice exposed to 4 Gy TBI, the result shows the same pattern with the ROS induction in the HSCs.

### 3.3 XMU‐MP‐1 improves the immune cells impairment induced by TBI 4 Gy

**FIGURE 3:**
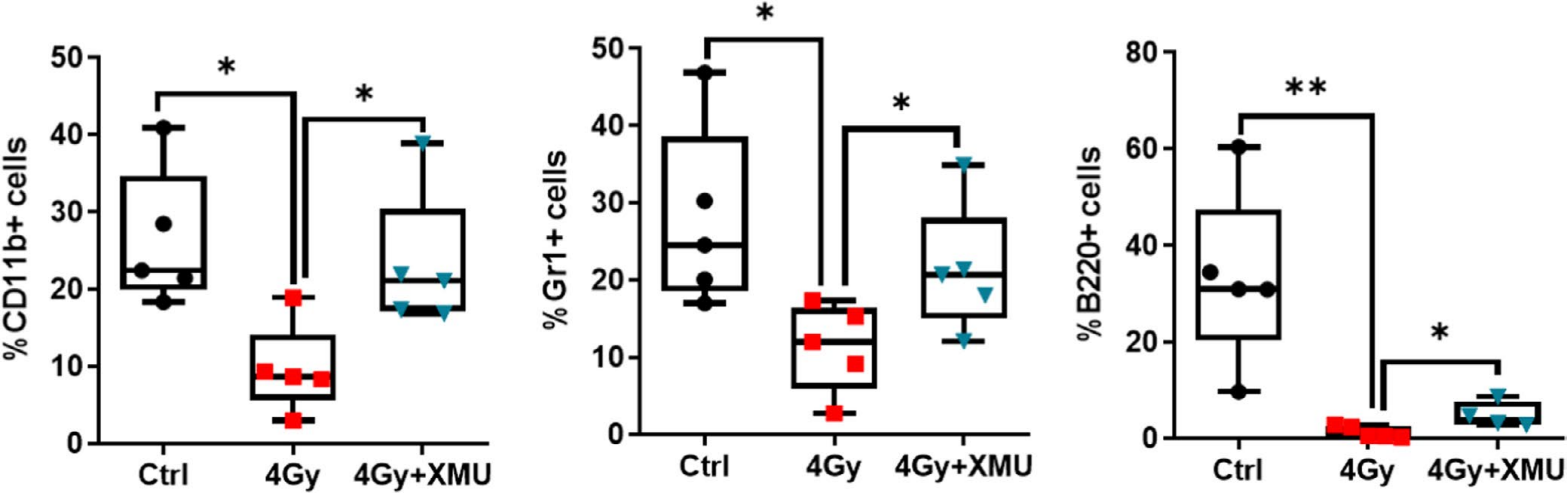
XMU‐MP‐1 relieves the immune cells impair induced by 4 Gy TBI. Mice were administrated with XMU‐MP‐1 for 7 days before being exposed to TBI 4 Gy. Bone marrow cells were harvested 9 days after IR and tested the cells proportion by flow cytometry. Results are expressed as Mean ± SD, *n* = 5 per group. (Significance: *p < 0.05, **p < 0.01 and ***p < 0.001)

The radiation exposure can suppress or weaken the immune system. CD11b was used to label myeloid lineage cells including monocytes, neutrophils and eosinophils. As Figure 3 shown that the proportion of CD11b+ cells depleted obviously (control, 26.3% ± 8.9% *vs*. 4 Gy 9.6% ± 5.8%, *p* < 0.05), while XMU‐MP‐1 pretreatment could rescue depletion of CD11b+ cells by increasing the proportion of it to 23.2% ± 9.0% (*p* < 0.05). Meanwhile, Gr‐1 labelled cells are comprised of Ly6C and Ly6G. Ly6C is commonly used to identify monocyte subtypes, while Ly6G is exclusively expressed on neutrophils. Population of Gr‐1+cells in bone marrow was also affected by 4 Gy TBI, while XMU‐MP‐1 could improve it significantly (control, 27.8% ± 11.8% *vs*. 4 Gy 11.3% ± 5.7% *vs*. 4 Gy+XMU 21.4% ± 8.4%, *p* < 0.05, *p* < 0.05). CD45R/B220 is used as a B cells marker from early pro‐, mature and activated B cells. The effect of XMU‐MP‐1 shows the same pattern in the B220+ cells, it decreased from 60.4% ± 18.0% to 1.3% ± 1.2% (*p* < 0.01) and recovered to 4.8% ± 2.7% (*p* < 0.05).

### 3.4 XMU‐MP‐1 relieves the intestinal injury induced by 9 Gy TBI

**FIGURE 4:**
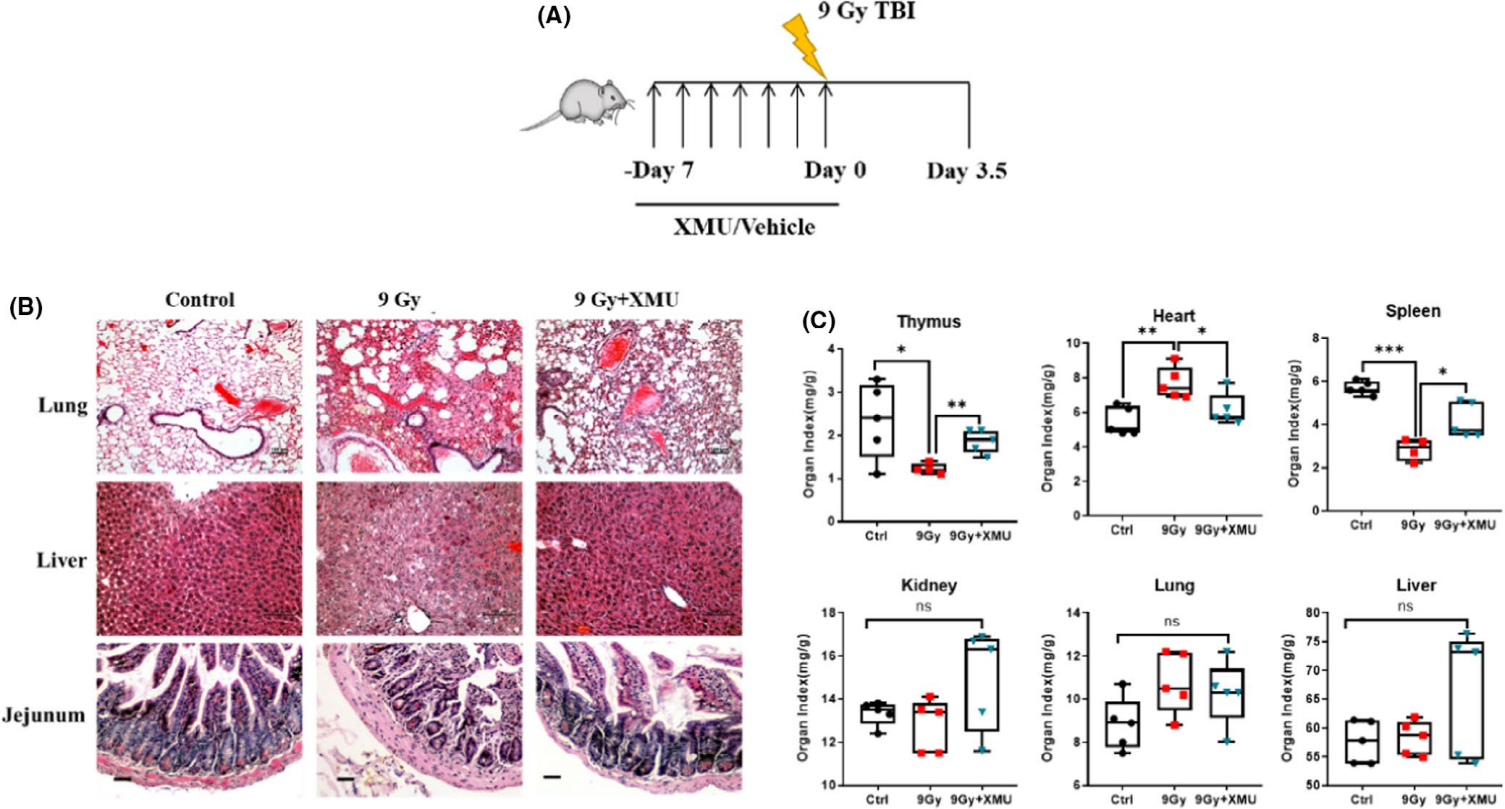
XMU‐MP‐1 protects the small intestinal injury induced by TBI. (A) Mice were administrated with XMU‐MP‐1 or vehicle for 7 days and then exposed to TBI 9 Gy. The samples were harvested 3.5 days after TBI; (B) The presentative image of haematoxylin and eosin staining of mice organs; Scale bar = 50 μm; (C) The organ index of mice in different groups. Organ Index = organ weight/body weight. Results are expressed as Mean ± SD, *n* = 5 per group. (Significance: *p < 0.05, **p < 0.01 and ***p < 0.001)

To determine the effect of XMU‐MP‐1 on the intestinal system exposed to irradiation, a higher dose of 9 Gy TBI was applied to mice. The radiation experiment was set up as the diagram Figure 4A shown. The results of haematoxylin and eosin staining (Figure 4B) show that the main pathological changes in the irradiated lung are inflammatory cells infiltration, oedema and bleeding; the porous cytoplasm, mild steatosis can be observed in the irradiated liver; the characteristic histological changes in the irradiated jejunum are markedly shorten villi, depletion of mucosal epithelial cells and crypts filled with deeply basophilic proliferative enterocytes. While the histopathological changes can be markedly recovered by XMU‐MP‐1 treatment.

The result (Figure 4C) illustrates that organ indexes of the thymus, spleen, heart showed various effect. The organ index of thymus as a target organ of irradiation reduced markedly from 2.3 ± 0.9 to 1.2 ± 0.1 (*p* < 0.05), while that of the XMU‐MP‐1 pretreated mice was improved to 1.9 ± 0.3 (*p* < 0.01). The organ indexes of spleen in the mice show the same pattern, which are 5.7 ± 0.3 (control), 2.9 ± 0.5 (9 Gy, *p* < 0.001) and 4.2 ± 0.8 (9 Gy+XMU‐MP‐1, *p* < 0.05). On the contrary, the organ indexes of heart in the irradiated mice were increased significantly (control, 5.5 ± 0.8; 9 Gy, 7.7 ± 0.9, *p* < 0.01) and XMU‐MP‐1 pretreatment remarkably diminished the elevation of it (9 Gy, 7.7 ± 0.9 vs 9 Gy+XMU‐MP‐1, 6.1 ± 0.9, *p* < 0.05). Meanwhile, the organ indexes of kidney, lung and liver did not show remarkable difference between groups.

### 3.5 XMU‐MP‐1 extends the survival days exposed to 7.5 Gy TBI

**FIGURE 5:**
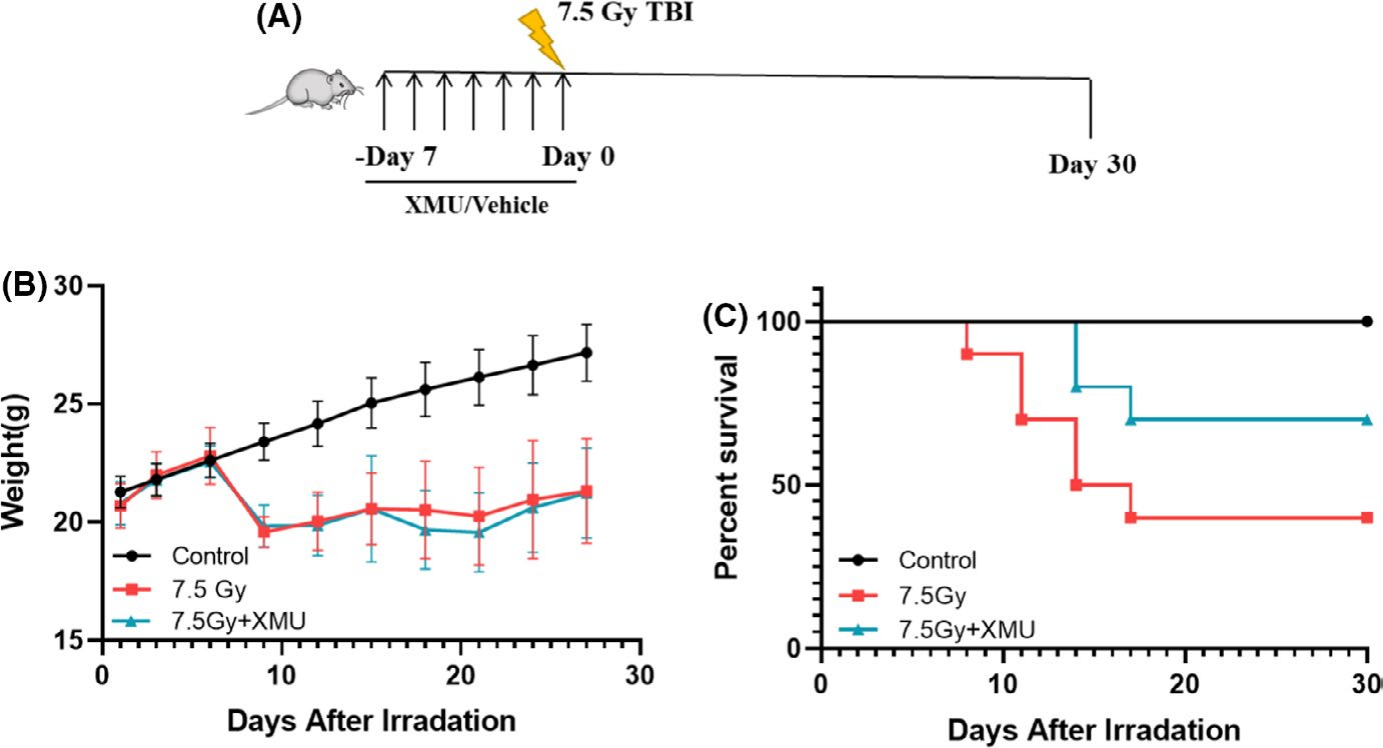
XMU‐MP‐1 increased survival of lethally irradiated mice. (A) Mice were administrated with XMU‐MP‐1 or vehicle for 7 days and then exposed to TBI 7.5 Gy. Mice were monitored for 30 days; (B) Weight of mice were recorded; (C) Survival curves of mice after TBI, *n* = 10

To evaluate the radioprotection of XMU‐MP‐1 to the acute radiation challenge, the lethal dose radiation of 7.5 Gy was applied to the mice with XMU‐MP‐1 treatment (shown in Figure 5A). And the results (Figure 5B) show that there existed a turning point of the body weight of irradiated mice at the 6th day after radiation. As Figure 5C shown, the average survival days of mice treated with 7.5 Gy+XMU is 25.5 days, longer than the mice treated with 7.5 Gy alone (19.2 days). While the body weight of the irradiated mice did not present the difference with XMU‐MP‐1 treatment, all the irradiated mice suffered from the weight loss starting at the 6th day. Although suffering with extreme weight loss, there were more mice survived in the XMU‐MP‐1 pretreated group. Therefore, XMU‐MP‐1 could provide a tolerance of the body weight loss challenge.

## 4 DISCUSSION

Ionizing radiation exposure on body can activate the boost of ROS, consequently induce a various reaction on DNA, protein and lipids. Double‐strand breaks (DSBs), although rare, are probably the most lethal mechanism and are commonly produced by ionizing radiation.^25^ Regarding the radiosensitivity, the quiescent and slowly dividing cells are less radiosensitive, like those constituting the nervous system, while cells with high proliferation rates are more vulnerable to radiation, including bone marrow, skin and epithelial cells of gastrointestinal tract. Radiation‐induced suppression of haematopoiesis and immune function has been one of the most life‐threatening consequences of radiation exposure.^26^ In order to assess the influence of radiation on the haematopoietic system, TBI 4 Gy as a sublethal dose was selected as the challenge of radiation exposure.^27^, ^28^ Our present data demonstrated that lymphocytes maintain an impair level after 9 days TBI 4 Gy, due to the irreversibly damaged haematopoietic stem cells no longer produce mature blood cells. Decline in WBC, RBC and platelets was identical events after irradiation exposure. While XMU‐MP‐1 pretreatment could remarkably rescue the suppression of haematopoietic system; meanwhile, the BMNCs, as a marker of bone marrow proliferation, were recovered to the level before radiation exposure. BMNCs counts presented the total nucleated cells population including haematopoietic stem cells and mesenchymal stem cells. XMU‐MP‐1 was reported as a reversible and selective MST1/2 inhibitor activating the downstream effector Yes‐associated protein and promoting cell growth.^29^ The Hippo pathway regulates the self‐renewal and differentiation of stem and progenitor cells and plays a key role in controlling size and regeneration. Previous studies revealed MST1/2 expressed in most organs as well as the haematopoietic system involved in hepatocellular sarcoma, intestinal adenocarcinoma and lymphoma.^30^ It was reported that the MST1/2 deficient mice suffer from the alternations in the steady‐stated HSC population in BM and impaired function HSCs under stress conditions.^31^ On the contrary, the bone marrow suppression induced by radiation exposure was alleviated by the MST1/2 inhibitor XMU‐MP‐1 treatment, which could be a consequence of MST1/2 rescuing the oxidative stress in HSCs.

Ionizing radiations represent the main source of DNA damage and ROS production in HSCs, indeed, increased ROS in HSCs initiates their differentiation and their exhaustion^32^, ^33^ and quiescent HSC with the lowest level of ROS have the highest haematopoietic reconstitution potential compared to ‘activated’ HSC harbouring higher ROS levels.^34^ Geng et al. documented that MST1/2 was vital for ROS‐mediated innate anti‐bacterial response.^18^ But on the other hand, MST1 inhibition mediated the cytoprotective action of mBM‐MSCs against H_2_O_2_‐induced oxidative stress injury.^34^ The underlying mechanisms involve autophagy activation and the Keap1/Nrf2 signal pathway. Our present data indicated that the increased ROS level of HSCs induced by TBI 4 Gy was diminished by XMU‐MP‐1 exposure; meanwhile, NOX4 expression level of HSCs was reduced significantly compared with irradiated mice. NOX4 as one of the NADPH oxidases (NOX) family proteins was involved in the production of ROS, showing increased expression after ionizing radiation.^35^ To reveal the details of NOX4 associated with the oxidative stress, the phosphorylated p‐38 and Nrf2 expression were evaluated by flow cytometry, and the results show that TBI‐induced increased ROS levels of HSCs through the NOX4/ROS/P38 MAPK signalling pathways. Meanwhile, the downregulated NOX4 expression by XMU‐MP‐1 explained the diminishment of ROS generation. The γ‐H2AX level of HSCs associated with the damaged DNA double helix presented the same pattern with ROS change, which could be the consequence of NOX4 expression inhibition by XMU‐MP‐1. Suffering from the impairment of immune system, the irradiated mice presented significantly lower number of granulocytes (Gr‐1+, CD11b+) and B cells (B220+) in peripheral blood compared with those found in vehicle‐treated controls (Figure 3). While the immune suppression induced by radiation could be attenuated by XMU‐MP‐1 markedly, the effect could partially benefit from the downregulation of NOX4/ROS/P38 MAPK pathway.

Despite the haematopoietic syndrome caused by radiation exposure, the gastrointestinal acute radiation syndrome can also be observed in clinic including pain, bloating, nausea and faecal urgency. As the result of Figure 4B exhibit characteristic morphological changes including decreased villi height and irregular, shortened microvilli (‘villus blunting’), as well as cytoplasmic vacuolization and detachment of epithelial and endothelial cells from their basement membrane. While XMU‐MP‐1 pretreatment a week prior to IR obviously prevented the morphological changes, especially the decrease in the counts and depth of crypts, indicating the benefical effect on the recovery of intestinal barrier function. The radiation‐induced lung injury encompassed two steps: the early step known as radiation pneumonitis, characterized by acute lung tissue inflammation, and the late step called radiation fibrosis, a clinical syndrome resulted from chronic pulmonary tissue damage.^36^ In the present study, the radiation pneumonitis is the main observation of the lung tissue in the mice at 3.5 days after exposed to TBI 9 Gy and the liver injury of radiation exposure was identified as hepatic steatosis and porous cytoplasm. XMU‐MP‐1 could alleviate the damage of lung and liver caused by ionizing radiation, which could be benefit from the protection of the haematopoietic and intestinal system. Spleen and thymus are two target organs of radiation injury, the reduced organ index of the irradiated spleen and thymus was recovered by XMU‐MP‐1 significantly, consistent with the effects of bone marrow failure and immunosuppression.

## 5 CONCLUSIONS

Numerous studies have reported that MST1/2 are important in cell proliferation, survival differentiation and are involved in diverse life process, including tissue homeostasis and tumour suppression. We demonstrated that MST1/2 inhibitor XMU‐MP‐1 mediated the radioprotection benefit of haematopoiesis and intestinal system against ionizing radiation‐induced oxidative stress injury. The underlying mechanisms are associated with inhibiting of the NOX4/ROS/P38 signalling pathway, however, the interaction between ROS regulation and Hippo signalling still need further investigation. These findings present that XMU‐MP‐1 could rescue the bone marrow failure and intestinal damage induced by radiation, and MST1/2 might be a potential target for radiation mitigator in clinic.

## CONFLICT OF INTEREST

The authors declare no conflicts of interest.

## AUTHOR CONTRIBUTIONS

**Xiaoliang Zhou:** Conceptualization (lead); Writing – original draft (lead). **Hao Wang:** Conceptualization (equal). **Deguan Li:** Methodology (equal); Resources (equal). **Naling Song:** Validation (equal). **Fujun Yang:** Visualization (equal). **Wenqing Xu:** Project administration (equal); Supervision (equal).

## Data Availability

Data openly available in a public repository that issues datasets with DOIs.
